# Clinical Images: Propylthiouracil‐induced antineutrophil cytoplasmic antibody–associated vasculitis with aortic wall thickening

**DOI:** 10.1002/acr2.11786

**Published:** 2025-01-12

**Authors:** Sho Ishigaki, Jun Kikuchi, Yuko Kaneko

**Affiliations:** ^1^ Division of Rheumatology, Department of Internal Medicine Keio University School of Medicine Tokyo Japan

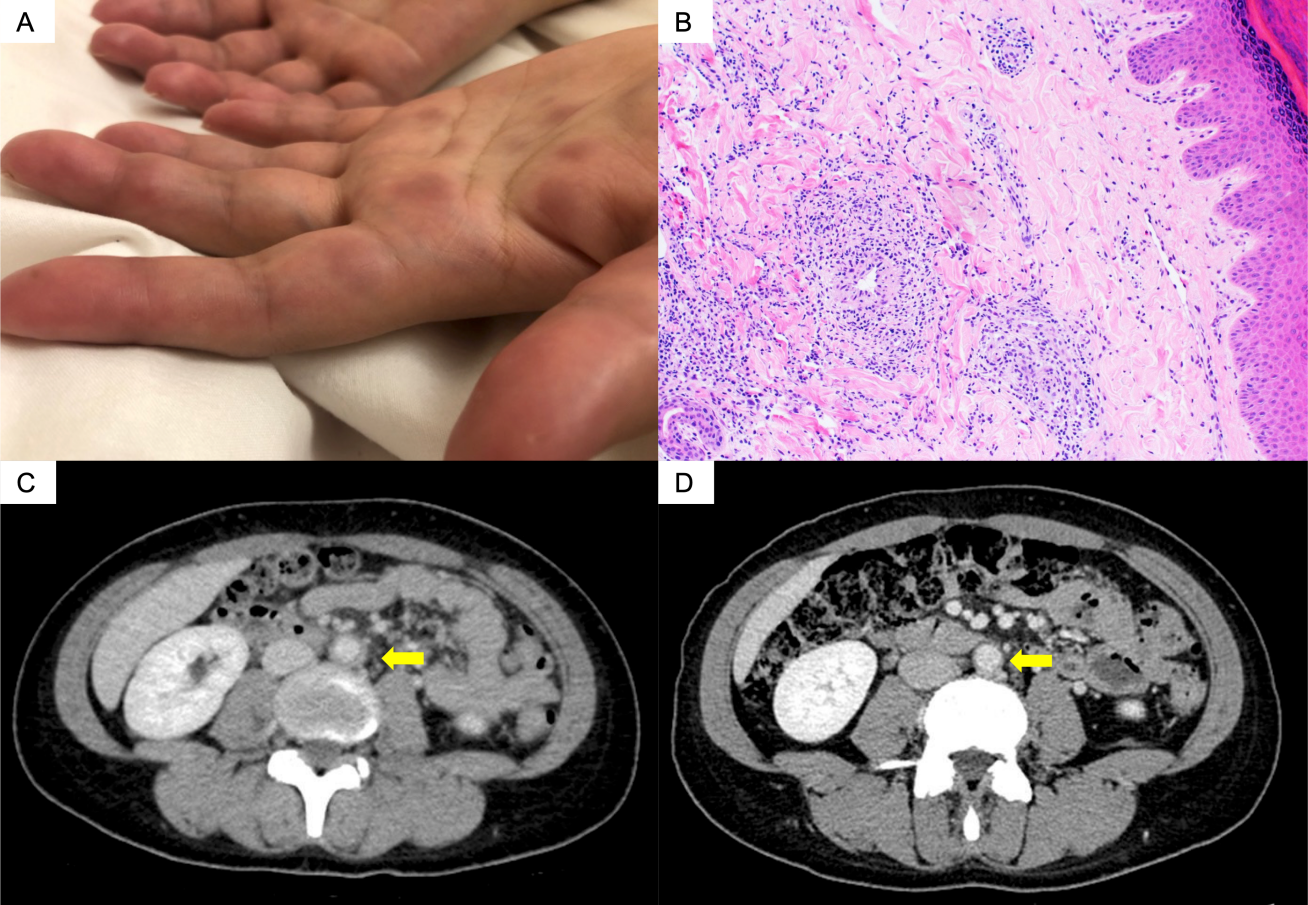

The patient, a 26‐year‐old woman, presented with arthritis. Two years before her consultation, she had been diagnosed with Graves disease and was started on methimazole. Her treatment was switched to propylthiouracil (PTU) during pregnancy for safety. After 10 months, she developed severe arthritis, fever, erythema, and scleritis. Blood tests revealed that her myeloperoxidase antineutrophil cytoplasmic antibody (ANCA) levels had risen above 300 U/mL, and a skin biopsy from the erythema showed small vessel vasculitis (A and B). Additionally, contrast‐enhanced computed tomography imaging revealed wall thickening around the aorta (C). Based on these findings, we diagnosed her with microscopic polyangiitis with aortic involvement.^1^ Although her initial symptoms partially improved after the cessation of PTU, her arthritis and fever worsened agan. We initiated high‐dose prednisolone and rituximab, which improved her symptoms rapidly. Three months later, we confirmed the disappearance of the wall thickening around the aorta (D). After one year, the dosage of prednisolone could be decreased to 7 mg/day. PTU is a known inducer of ANCA‐associated vasculitis (AAV) due to impact on neutrophil extracellular trap formation.^2^ The lack of lung or renal involvement, young age, and the two‐year period from the start of PTU therapy to symptom onset suggest a high likelihood of PTU‐induced AAV.^3^ Although large vessel involvement could be observed in cases of primary AAV, this is the first reported case of PTU‐induced AAV with periaortitis.^4^

## Supporting information


**Disclosure Form**:

## References

[1] Suppiah R , Robson JC , Grayson PC , et al; DCVAS Study Group . 2022 American College of Rheumatology/European Alliance of Associations for Rheumatology classification criteria for microscopic polyangiitis. Arthritis Rheumatol 2022;74(3):400–406. 35106973 10.1002/art.41983

[2] Nakazawa D , Tomaru U , Suzuki A , et al. Abnormal conformation and impaired degradation of propylthiouracil‐induced neutrophil extracellular traps: implications of disordered neutrophil extracellular traps in a rat model of myeloperoxidase antineutrophil cytoplasmic antibody‐associated vasculitis. Arthritis Rheum 2012;64(11):3779–3787. 22777766 10.1002/art.34619

[3] Chen M , Gao Y , Guo XH , et al. Propylthiouracil‐induced antineutrophil cytoplasmic antibody‐associated vasculitis. Nat Rev Nephrol 2012;8(8):476–483. 22664738 10.1038/nrneph.2012.108

[4] Kaymakci MS , Elfishawi MM , Langenfeld HE , et al. Large vessel involvement in antineutrophil cytoplasmic antibody‐associated vasculitis. Rheumatology (Oxford) 2024;63(6):1682–1689. 37672018 10.1093/rheumatology/kead467

